# Depo-Provera® Treatment Does Not Abrogate Protection from Intravenous SIV Challenge in Female Macaques Immunized with an Attenuated AIDS Virus

**DOI:** 10.1371/journal.pone.0009814

**Published:** 2010-03-23

**Authors:** Meritxell Genescà, Michael B. McChesney, Christopher J. Miller

**Affiliations:** 1 Center for Comparative Medicine, University of California Davis, Davis, California, United States of America; 2 California National Primate Research Center, University of California Davis, Davis, California, United States of America; 3 Department of Pathology, Microbiology and Immunology, School of Veterinary Medicine, University of California Davis, Davis, California, United States of America; 4 Division of Infectious Diseases, School of Medicine, University of California Davis, Davis, California, United States of America; University of Toronto, Canada

## Abstract

**Background:**

In a previous study, progesterone treatment of female monkeys immunized with live, attenuated SHIV89.6 abrogated the generally consistent protection from vaginal simian immunodeficiency virus (SIV) challenge. The mechanisms responsible for the loss of protection remain to be defined. The objective of the present study was to determine whether Depo-Provera® administration alters protection from intravenous SIV challenge in SHIV-immunized female macaques.

**Methods and Findings:**

Two groups of female macaques were immunized with attenuated SHIV89.6 and then challenged intravenously with SIVmac239. Four weeks before challenge, one animal group was treated with Depo-Provera®, a commonly used injectable contraceptive progestin. As expected, SHIV-immunized monkeys had significantly lower peak and set-point plasma viral RNA levels compared to naïve controls, but in contrast to previously published findings with vaginal SIV challenge, the Depo-Provera® SHIV-immunized animals controlled SIV replication to a similar, or even slightly greater, degree than did the untreated SHIV-immunized animals. Control of viral replication from week 4 to week 20 after challenge was more consistent in the progesterone-treated, SHIV-immunized animals than in untreated, SHIV-immunized animals. Although levels of interferon-γ production were similar, the SIV-specific CD8^+^ T cells of progesterone-treated animals expressed more functions than the anti-viral CD8^+^ T cells from untreated animals.

**Conclusions:**

Depo-Provera® did not diminish the control of viral replication after intravenous SIV challenge in female macaques immunized with a live-attenuated lentivirus. This result contrasts with the previously reported effect of Depo-Provera® on protection from vaginal SIV challenge and strongly implies that the decreased protection from vaginal challenge is due to effects of progesterone on the genital tract rather than to systemic effects. Further, these results demonstrate that the effects of hormonal contraceptives on vaccine efficacy need to be considered in the context of testing and use of an AIDS vaccine.

## Introduction

In the simian immunodeficiency virus (SIV) model of AIDS, only live-attenuated viruses consistently confer reliable protection against intravaginal virus inoculation [1], [2]. Immunizing rhesus macaques with attenuated simian/human immunodeficiency virus (SHIV) 89.6 protects 60% of the animals from uncontrolled SIVmac239 virus replication and progression to AIDS after intravaginal challenge [1]–[3]. However, treatment with progesterone prior to SIV challenge dramatically decreases the efficacy of this live-attenuated vaccine strategy [4]. Although SHIV 89.6 infection is persistant in the immunized animals, in this model vaccine failure is due to uncontrolled SIV replication and not due to replication of the vaccine virus [3]. Elucidating the mechanisms by which progesterone decreases attenuated SHIV-mediated protection from vaginal SIV challenge may provide insight into the immune mechanisms involved in protection in this model.

Because HIV infection affects mainly women of reproductive age, it is important to consider the impact of hormonal contraception on HIV transmission and progression of HIV infection [5]–[8]. Moreover, Depo-Provera®, a contraceptive progestin, is used in the SIV/macaque vaginal transmission model to synchronize menstrual cycles and increase SIV transmission efficiency [9], a strategy that may affect the results of SIV vaccine experiments [10]. Administration of Depo-Provera® to macaques in the commonly used dosage, 30 mg, leads to epithelial thinning [9], but genital tract epithelial thinning has not been found in women using this contraceptive at a lower dose [10], [11]. Thus, it is unclear if this mechanism of enhancing susceptibility to SIV infection is relevant to women.

In addition to thinning the vaginal mucosal of rhesus macaques, sex steroid hormones have many complex and undefined effects on the immune system that might modify S/HIV transmission and affect disease progression [10]. Progesterone induces cell-mediated immunosupression, which may help to maintain a successful pregnancy [10], [12]–[16]. Progesterone increases the recruitment of inflammatory cells into the genital mucosa, which could potentially promote HIV-1 transmission [17]. Conversely, progesterone can reduce pro-inflammatory cytokine and chemokine production [18]–[20]. Which, if any, of these effects contribute to the decreased protection from vaginal SIV challenge in SHIV-immunized rhesus macaques treated with Depo-Provera® is unknown.

The objective of the present study was to determine if SHIV-immunized female rhesus macaques treated with Depo-Provera® are protected after intravenous (IV) SIV challenge. We reasoned that if the levels of protection from IV and vaginal SIV challenge are equivalent in SHIV-immunized macaques treated with Depo-Provera®, then systemic, rather than vaginal mucosal, effects of progesterone account for the reduced protection from vaginal SIV challenge after Depo-Provera® treatment [4]. We found that treatment with Depo-Provera® did not decrease vaccine efficacy compared to untreated, SHIV-immunized animals, and unexpectedly, it may have increased the proportion of animals within the Depo-Provera®- treated group that control viral replication. After peak viremia, the difference in plasma viral RNA (vRNA) levels between the Depo-Provera®-treated group and the unimmunized, control group was more significant than the difference in the plasma vRNA levels between the untreated, SHIV-immunized animals and the unimmunized group (p<0.01 vs p<0.05). Vaccine-mediated protection in the Depo-Provera® SHIV-immunized animals was associated with an increased number of SIV specific-CD8^+^ T cells within an environment of regulated immune activation.

## Methods

### Animals

Multiparous, normally cycling female rhesus macaques (*Macaca mulatta*) were housed at the California National Primate Research Center in accordance with the regulations of the American Association for Accreditation of Laboratory Animal Care standards. The experiments were approved by the Institutional Animal Care and Use Committee of the University of California, Davis. All animals were negative for antibodies to HIV-2, SIV, simian type-D retrovirus, and simian T cell lymphotropic virus type1 at the time the study was initiated. To minimize discomfort, animals were anesthetized with ketamine hydrochloride (10 mg/Kg; Parke-Davis, Morris Plains, NJ) or 0.7 mg/kg tiletamine HCl and zolazepan (Telazol, Fort Dodge Animal Health, Fort Dodge, IA) injected intramuscularly for all procedures.

### SHIV89.6 immunization, Depo-Provera® treatment and SIV challenge

Eleven macaques were immunized by IV inoculation with live, attenuated SHIV89.6 [1]. Six months after SHIV 89.6 inoculation, and 4 weeks before challenge with SIV, a single 30 mg dose of Depo-Provera® [4] was administered by intramuscular injection to 6 randomly selected macaques. Hereafter, these 6 macaques are referred as “Depo-Provera® SHIV macaques” and the 5 untreated, SHIV-immunized macaques are referred as “SHIV macaques”. Seven unvaccinated control macaques referred as “naïve controls” were challenged IV with SIVmac239 contemporaneously with the 11 SHIV-immunized macaques. A stock of pathogenic SIVmac239 was used as previously described [2] and contained approximately 10^5^ TCID50/ml. All of the animals were given a single IV inoculation containing 10^3^ TCID50 of the SIVmac239 stock in 1 ml of sterile saline. Approximately, five months after IV SIVmac239 challenge, all the monkeys were necropsied and blood and tissues were collected.

### PBMC Isolation

Peripheral blood mononuclear cells (PBMC) were isolated as described [2].

### RNA isolation and cytokine mRNA levels

Total RNA was isolated with Trizol (Invitrogen, Carlsbad, CA) according to the manufacturer's instructions from cryopreserved PBMC samples. RNA samples were DNase treated with DNA-free (Ambion) for 1 h at 37°C. cDNA was prepared using random hexamer primers (Amersham-Pharmacia Biotech, Inc., Piscataway, NJ) and superscript III reverse transcriptase (Invitrogen). Cytokine mRNA levels were determined by real-time PCR as described previously [1].

### Plasma vRNA Measurement

Plasma samples were analyzed for vRNA by a quantitative branched DNA assay as previously described [2]. Viral titers in plasma samples are reported as vRNA copy numbers per ml of plasma. The quantitation limit of this assay was 125 vRNA copies/ml plasma.

### SIV-specific interferon-γ ELISPOT Assay

The number of interferon (IFN)-γ secreting cells in cryo-preserved PBMC responding to a SIVmac239 Gag p27 peptide pool was determined using a monkey-specific IFN-γ ELISPOT kit (U-CyTech, Utrecht University, Utrecht, Netherlands) as previously described [1], [21].

### SIV-specific T cell Proliferation Assay

Proliferation of PBMC in response to aldrithol-2-inactivated SIVmac239 (provided by J. Lifson, SAIC Frederick, Bethesda, MD) was measured by 3-H thymidine incorporation as previously described [1], [22]. A stimulation index (SI), calculated as the mean counts per minute of replicate antigen wells divided by the mean counts per minute of control wells, was scored positive if >2.0.

### Flow cytometric analysis of cell populations in blood

The percentage of CD3^+^CD4^+^ and CD3^+^CD8^+^ T cells, natural killer and CD20^+^ B cells, within the lymphocyte gate of PBMC samples, was determined as described [21].

### Intracellular cytokine staining of SIV-specific T cells

Cryopreserved PBMC were thawed, rested overnight and stimulated with a p27 Gag-peptide pool at 5 µg/ml, or with Gag epitope-peptides (CM9 or GY9) at 1 µg/ml, as described [23]. Background controls contained co-stimulatory antibodies and dimethyl sulfoxide, and a positive control was stimulated with staphylococcal enterotoxin B. Intracellular staining for Ki67 and caspase 3 was performed as described [23].

### Measurement of TNF-α and TGF-β in plasma

Tumor necrosis factor (TNF-α) and transforming growth factor (TGF-β) levels in EDTA plasma samples were determined using commercial ELISA kits according to the manufacturer's instructions (Quantikine®, R&D Systems, Minneapolis, Minn.).

### Statistical Analysis

The results of statistical analyses are reported as the mean and the standard error of the mean for each group. Student's t test was performed for single time-point comparisons of Depo-Provera® treated SHIV-immunized and SHIV-immunized animals. The non-parametric Friedman test for repeated measures, with Dunn's multiple comparisons post-hoc test, was used to analyze differences among the three groups over the entire post-challenge period. To determine if the cummulative levels of plasma vRNA, of proliferative response or of spot-forming cells among the 3 monkey groups were significantly different over the post-challenge phase, the median area under the curve (AUC) for each animal group was computed and a Kruskal-Wallis test was applied. To compare the median AUC between each monkey group, Dunn's multiple comparisons post-hoc test was used. The Spearman rank correlation test was used to determine the correlation between mRNA gene expression and viral load. All the above calculations were done using Prism 4.0 software (Graph Pad Inc.) and a Macintosh G5 computer (Apple Inc. Cupertino, CA). *P* values of <0.05 were considered significant.

## Results

### Progesterone does not abrogate control of virus replication and preserves CD4^+^ T cells after IV SIVmac239 challenge

**Figure 1:**
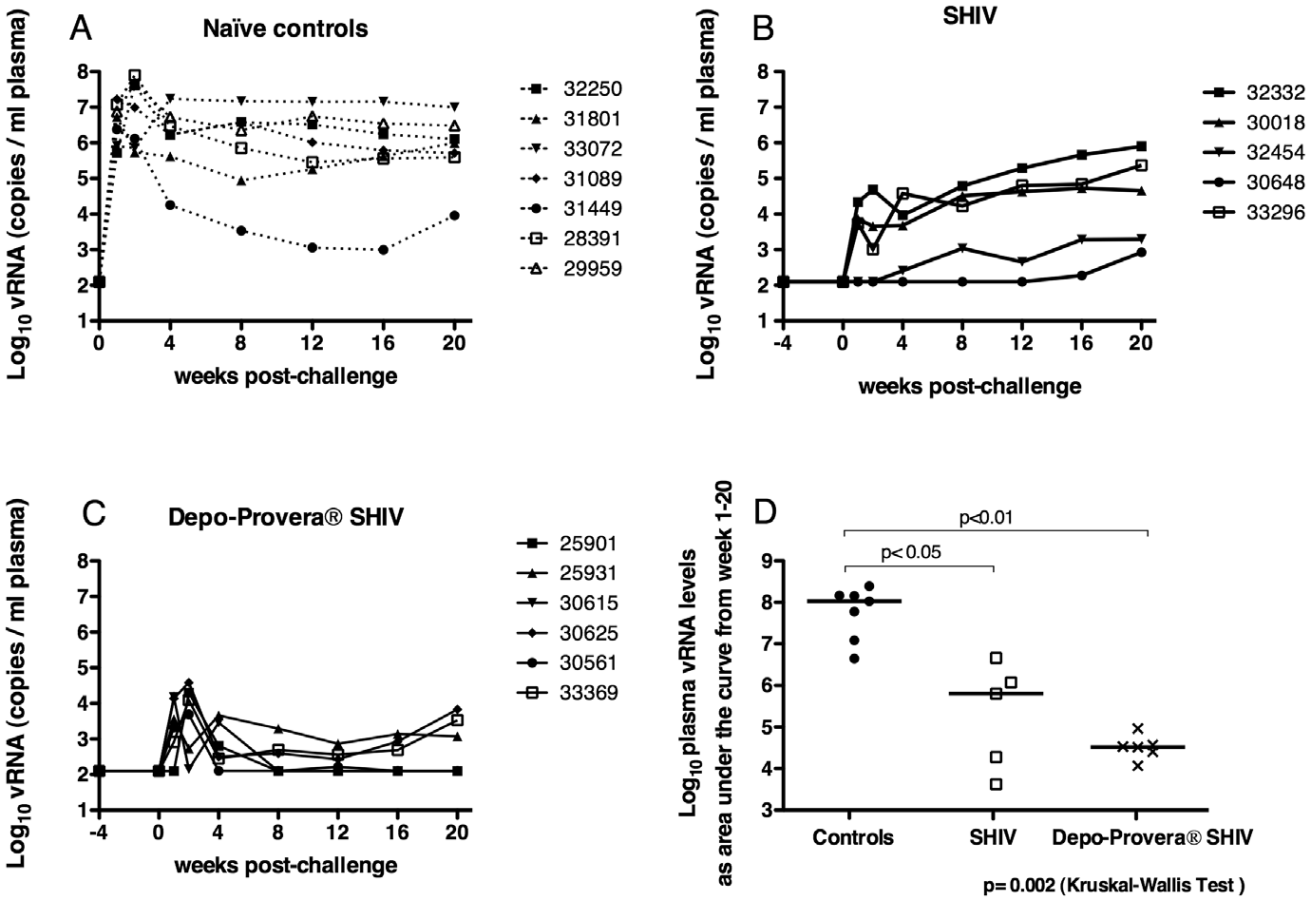
Plasma vRNA levels after IV challenge with SIVmac239. (A) unimmunized macaques, (B) SHIV-immunized macaques and (C) progesterone-treated, SHIV-immunized macaques. In panel (D), cumulative vRNA levels over the 20 weeks of observation were transformed into areas under the curve (AUC) and the median AUC of the 3 groups of animals were compared. The Kruskal-Wallis test and a pair-wise comparison between the 2 groups of immunized animals and the unimmunized group using Dunn's multiple comparisons test were performed.

Eleven female rhesus macaques were inoculated with SHIV89.6 and at 24 weeks after immunization (four weeks prior to IV SIVmac239 challenge), Depo-Provera® was administered to 6 randomly selected animals. Of note, there was no change in SHIV plasma vRNA levels after treatment (Figure 1). At 1–2 weeks post-challenge (PC), all 7 control animals had high peak plasma vRNA levels of 10^7^ vRNA copies/ml, which decreased to set point at 8–12 weeks PC (Figure 1A). Nine of 11 SHIV89.6 immunized animals had vRNA detected in plasma collected from 1–4 weeks PC, and there was no difference between the two immunized groups (*p = *0.79, two-tailed unpaired T test; Figure 1B and C). However, at 4 weeks PC, SHIV-immunized monkeys developed very distinct patterns of viral replication. Animals defined as “protected” controlled viral replication (set point plasma vRNA levels <10^4^ vRNA copies/ml) and had stable CD4^+^ T cells counts. Animals defined as “unprotected” had plasma vRNA set point levels >10^4^ vRNA copies/ml and a decline in CD4^+^ T cells. Based on these criteria, 100% (6/6) of the Depo-Provera® SHIV macaques were protected from uncontrolled virus replication through 20 weeks PC. In contrast, only 40% (2/5) SHIV macaques were protected from IV challenge with SIV. Although in this study we did not distinguish which virus replicated in the immunized macaques after challenge or whether recombination occurred, a similar study using the same model has clearly shown that only the challenge virus, SIVmac239, accounts for uncontrolled viral replication after challenge [3].

Using the plasma vRNA data for each animal, vRNA levels over the entire post-challenge period were transformed into an AUC value. The median plasma vRNA AUC for each of the 3 animal groups were significantly different (*p = *0.002, Kruskal-Wallis test) from one another (Figure 1D). While the SHIV macaque group had a significantly lower (*p*<0.05, Dunn's multiple comparison post-hoc test) mean plasma vRNA AUC value than the naïve control group, the mean vRNA AUC value of the Depo-Provera® SHIV group was even lower compared to the naïve control group (*p*<0.01, Dunn's multiple comparison post-hoc test) (Figure 1D). These AUC analyses were further broken down to distinguish peak and set point viremia. At peak viremia (0 to 4 week PC) both immunized groups had significantly lower viral load compared to the naïve control macaques (*p = *0.0023 in Kruskal-Wallis test, *p*<0.01 for both SHIV groups in Dunn's multiple comparison post-hoc test), but the two immunized groups were not different from each other. Further, from week 8 to 20 PC, only the Depo-Provera® SHIV monkeys had significantly lower viral loads compared to naïve control macaques (*p = *0.0039, data not shown).

**Figure 2:**
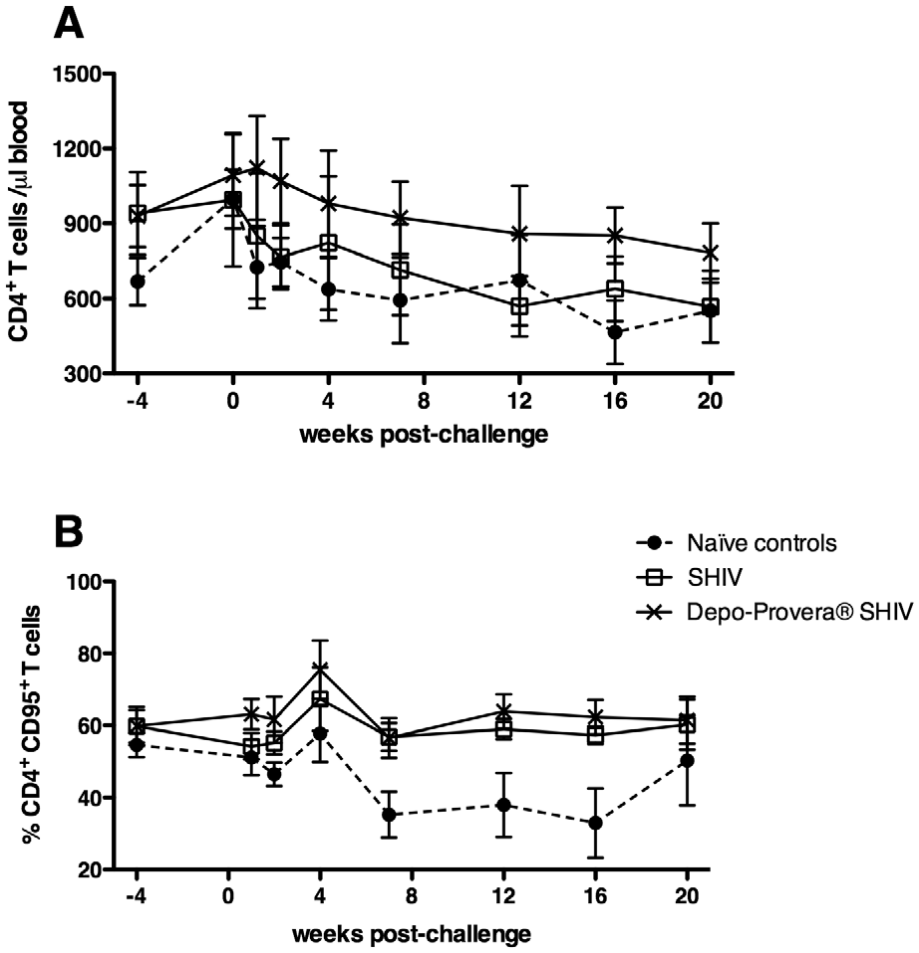
Changes in peripheral CD4^+^ T cell populations after IV SIVmac239 challenge. (A) Number of CD4^+^ T cells, and (B) the percentage of CD4^+^ CD95^+^ T cells in blood. (λ) Naïve control macaques (n = 7), (○) SHIV-immunized macaques (n = 5) and (5) Depo-Provera® SHIV-immunized macaques (n = 6). The number of CD4^+^ T cells (p = 0.0007) and the percentages of CD4^+^ CD95^+^ T cell (p<0.0001) were significantly higher (Friedman test) in the Depo-Provera®-treated, SHIV-immunized macaques than in the naïve control group.

The absolute number of CD4^+^ T cells in blood over the entire post-SIV challenge time period differed among the 3 animal groups (*p = *0.0007, Friedman test, Figure 2A) as Depo-Provera® SHIV monkeys had significantly higher mean CD4^+^ T cells than the naïve control group (*p*<0.01; Figure 2A), while the SHIV group did not. Additionally, after challenge, the frequency of circulating memory CD95^+^ CD4^+^ T cells was significantly higher in the Depo-Provera® SHIV monkeys but not in the untreated SHIV monkeys compared to the naïve control group (*p*<0.0001, Friedman test; Figure 2B). Thus, as observed for control of viral replication, no differences in CD4^+^ T cell number were attributable to progesterone treatment in these analyses. However, when compared to naïve control monkeys, Depo-Provera® treated macaques maintained significantly higher levels of memory and total CD4^+^ T cells, while in the SHIV group, there was only a trend.

### SIV-specific T cell responses were stronger and more polyfunctional in the Depo-Provera®-treated, SHIV-immunized than in the SHIV-immunized macaques after SIV challenge

**Figure 3:**
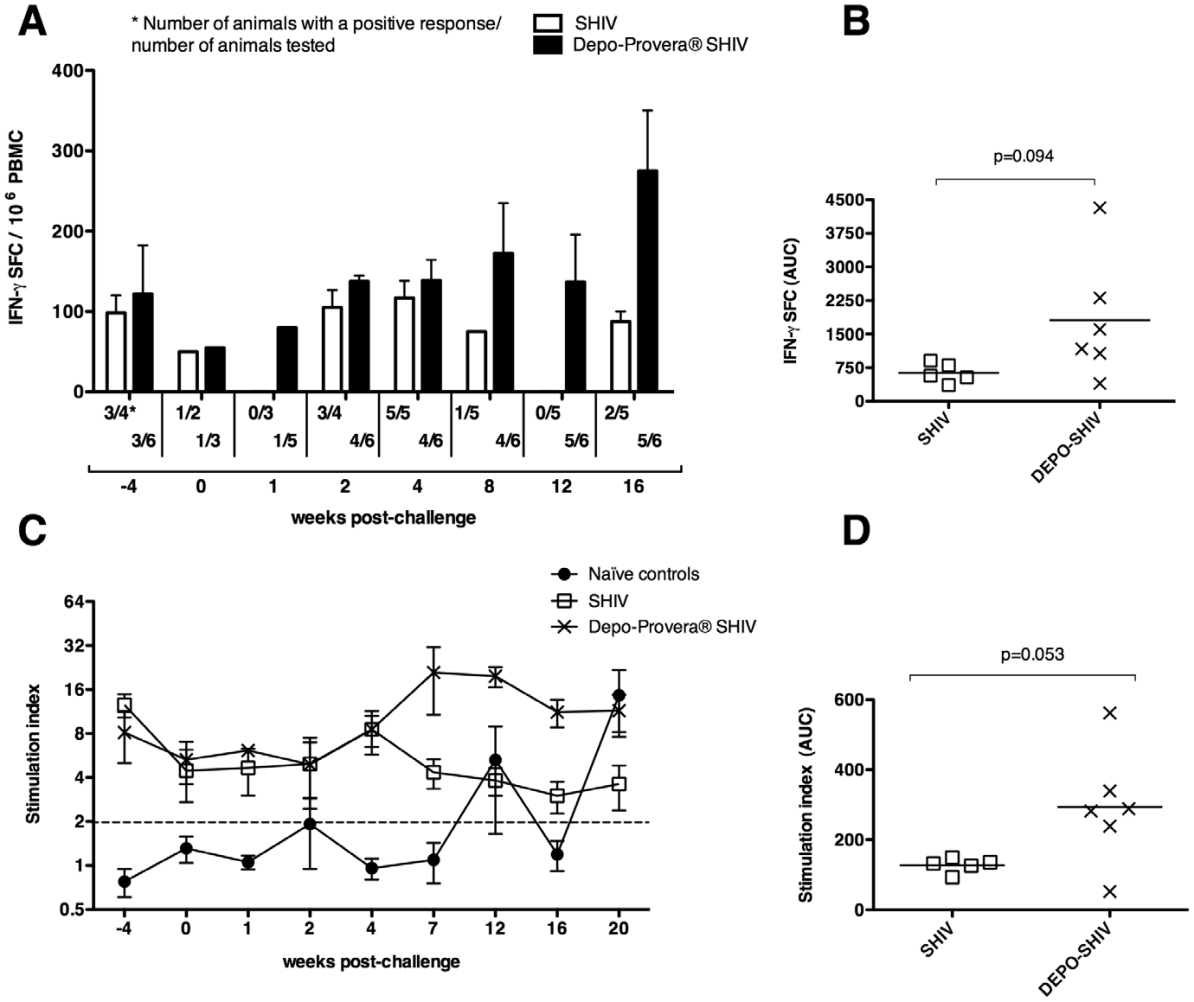
SIV-specific T cell responses after IV SIVmac239 challenge. (A) Mean number of SIV Gag p27 interferon (IFN)-γ secreting cells in PBMC before and after challenge. (B) Median area under the curve for the total IFN-γ secreting cells in each group from week 2 to 16 PC, the time-points for which samples were available. (C) Mean SIV-specific T cell proliferative responses in PBMC expressed as the stimulation index (SI) before and after SIV challenge. See *Methods* for details. (D) Median area under the curve for the cumulative SI in each group from week -4 to 20 PC. (λ) Naïve control macaques (n = 7), (○) SHIV-immunized macaques (n = 5) and (5) Depo-Provera® SHIV-immunized macaques (n = 6). P values of a T test are indicated.

SIV-specific T cell proliferative responses and IFN-γ spot forming cells were assessed in PBMC samples (Figure 3). SIV Gag-specific IFN-γ ELISPOT responses were detected in the 2 SHIV-vaccinated groups from the day of progesterone treatment until 16 week PC (Figure 3A), and when the IFN-γ spot forming cell counts for each animal, from week 2 to 16 PC, were aggregated into an AUC value, a non-significant trend toward higher responses in the progesterone-treated group was detected (Figure 3B, *p* = 0.094, T test). After week 4 PC, SIV-specific T cell proliferative responses were highest in the Depo-Provera® SHIV monkeys (Figure 3C) and only this group had significantly higher T cell proliferative responses than the naïve control group overtime (*p = *0.03, Friedman test). If each animal's T cell proliferative responses were aggregated to form an AUC, the Depo-Provera® SHIV group tended do have a higher cumulative proliferative response compared to the SHIV group (Figure 3D, *p = *0.053, T test).

**Figure 4:**
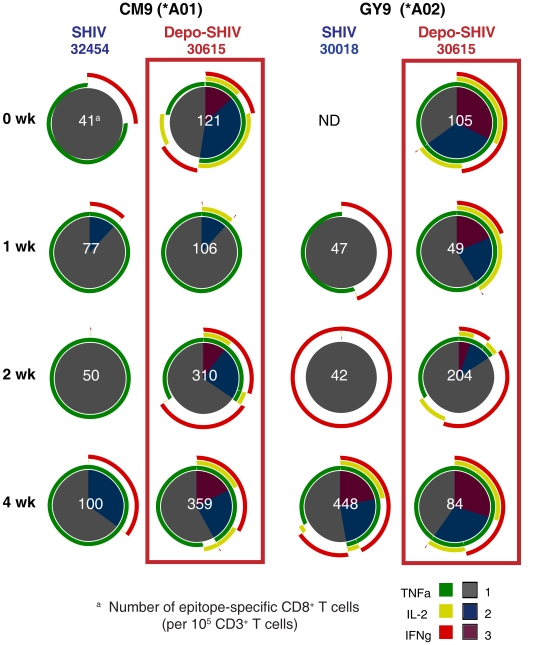
Frequency and functional capacity of Gag CM9- and GY9-specific CD8^+^ T cells of SHIV-immunized animals after IV SIVmac239 challenge. SIV-specific CD8^+^ T cell responses in cryopreserved PBMC from a *Mamu*-A*01 positive animal and a *Mamu*-A*02 positive animal in each vaccinated group are shown as pie charts. The number of positive SIV-specific T cells normalized to 10^5^ CD3^+^ T cells is shown for each response as the white number in the center of the pie chart. Each portion of a pie chart indicates the percentage of SIV-specific T cells that responded with one, two, or three functions; and the colored arcs around the pie show the cytokine or combination of cytokines comprising each response. ND not done, as samples were not available.

There were no significant differences in strength or pattern of SIV p27-Gag specific CD8^+^ T cell responses in PBMC of the 2 SHIV-immunized groups as measured by intracellular cytokine staining. However, in SHIV-immunized monkeys that were MHC-typed as Mamu-A*01^+^ (one in each group) or Mamu-A*02^+^ (one in each group), the frequency of CD8^+^ T cells responding to immunodominant SIV Gag epitopes (CM9 for Mamu-A*01 or GY9 for Mamu-A*02) was up to 6 times higher in the Depo-Provera® SHIV monkeys than in the SHIV monkeys during acute infection (Figure 4). Further the SIV-specific T cell responses in the Depo-Provera® -treated animals were comprised of poly-functional T cells, while the untreated, SHIV-immunized macaques had mono-functional T cells secreting IFN-γ or TNF-α, with rare IL-2 secreting CD8^+^ T cells (Figure 4).

### Depo-Provera® treatment increased T cell activation and apoptosis before SIV challenge but reduced T cell proliferation and apoptosis after SIV challenge

**Figure 5:**
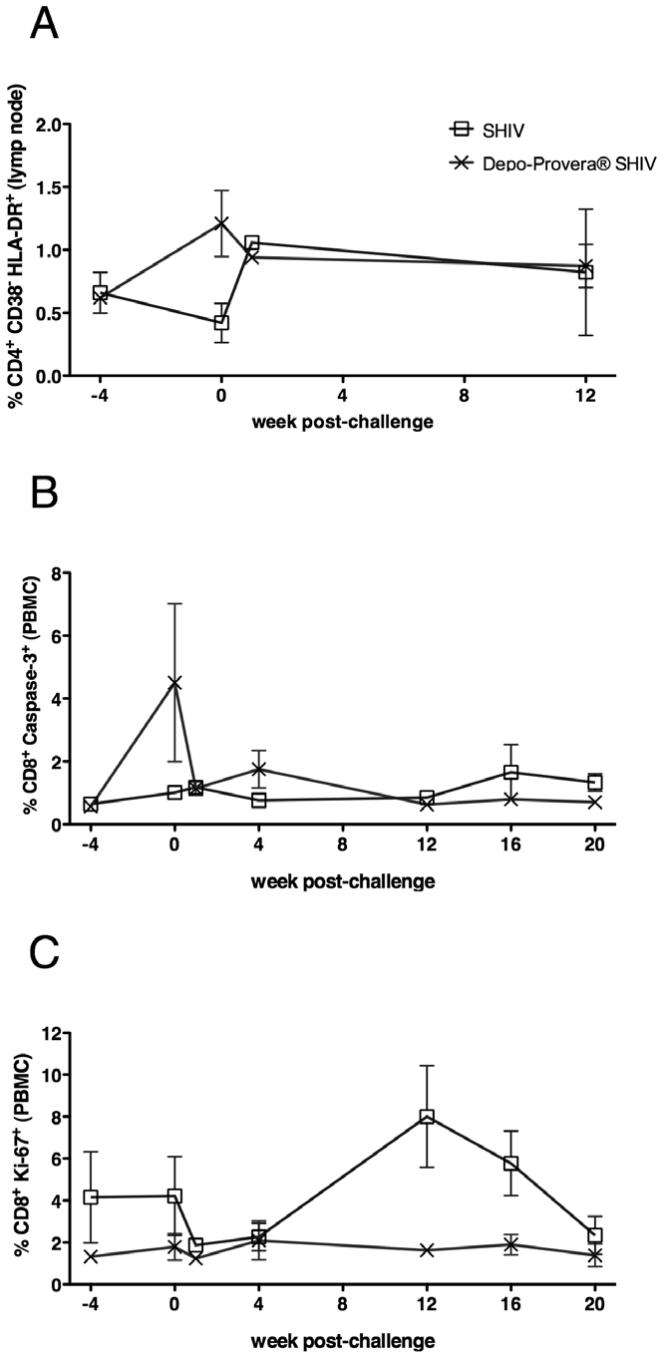
Frequencies of activated T cells after progesterone treatment and IV SIVmac239 challenge. A) Percent of CD3^+^ CD4^+^ T cells expressing CD38 but not HLA-DR in PBMC. B) Percent of CD3^+^ CD8^+^ T cells expressing CD38 but not HLA-DR in PBMC. D) Percent of CD3^+^ CD8^+^ T cells expressing Ki-67^+^ in PBMC. E) Percent of CD3^+^ CD8^+^ T cells expressing caspase-3^+^ in PBMC. (○) SHIV-immunized macaques (n = 5) and (5) Depo-Provera®-treated, SHIV-immunized (n = 6).

There was a significant increase in frequency of HLA-DR^+^CD38^-^CD4^+^ T cells in the lymph nodes (LN) of the Depo-Provera® SHIV monkeys between the day of progesterone treatment and the day of SIV challenge (from a mean±SEM of 0.62±0.04 to 1.21±0.26; *p = *0.032; Figure 5A). Further, the frequencies of activated, caspase-3^+^ CD8^+^ T cells in PBMC significantly increased between the day of treatment and the day of challenge in the Depo-Provera® SHIV monkeys (from 0.56±0.1 to 4.5±2.5; *p = *0.041; Figure 5B).

At 1 and 2 weeks PC, both SHIV-immunized groups had lower frequencies of CD38^+^CD8^+^ T cells in PBMC (∼55%) compared to the naïve control group (>70%, *p<*0.005 for the Depo-Provera® SHIV group, *p<*0.02 for the SHIV group; data not shown). At 1 week PC, the HLA-DR^+^CD38^−^ subsets of CD4^+^ and CD8^+^ T cells in PBMC were significantly increased in both SHIV groups compared to the naïve control group (∼1% vs <0.5%, *p<*0.05 in each subset from each group; data not shown). However at 2 weeks PC, these HLA^−^DR^+^CD38^−^ T cell subsets remained significantly higher only in the Depo-Provera® SHIV group compared to the naïve control group (*p = *0.009 for CD8^+^ T cells and *p = *0.0001 for CD4^+^ T cells; data not shown).

Further, after challenge, the frequencies of Ki-67^+^ CD4^+^ T cell in PBMC and axillary LN were similar in the two SHIV immunized groups, although the frequency tended to be lower in the Depo-Provera® treated monkeys. By 12 weeks PC, there was a significantly increased percentage of Ki-67^+^ CD8^+^ T cells in the PBMC (1.6±0.2 vs 8.0±2.4; *p = *0.018; Figure 5C) and axillary LN (1.8±0.2 vs 3.5±1.0; *p = *0.045) of the SHIV macaques compared to the Depo-Provera® SHIV macaques, in which Ki-67^+^ CD8^+^ T cells remained at a low level. At 12 weeks PC, in the axillary LN of SHIV macaques there was a significant increase in the frequency of caspase-3^+^ CD8^+^ T cells compared to Depo-Provera® treated monkeys (1.9±0.4 vs 5.2±1.1; *p = *0.009). Finally, the frequency of Ki67^+^ caspase-3^+^ T cells in tissues of SHIV-immunized monkeys at 12 weeks PC were very similar to the levels detected in naïve controls and significantly higher compared to the Depo-Provera® SHIV monkeys (*p = *0.008 for CD8^+^ and *p = *0.034 for CD4^+^ T cells; data not shown). Thus, while progesterone treatment induced T cell activation and turnover before and immediately after SIV challenge, it was soon restricted. T cell activation, proliferation and apoptosis was limited in the hormone-treated group after challenge, while at 8–12 weeks PC the untreated monkeys with high levels of viral replication had elevated levels of T cell activation and turnover.

### Increased regulatory T cell frequencies in Depo-Provera®-treated monkeys before and after SIV challenge

**Figure 6:**
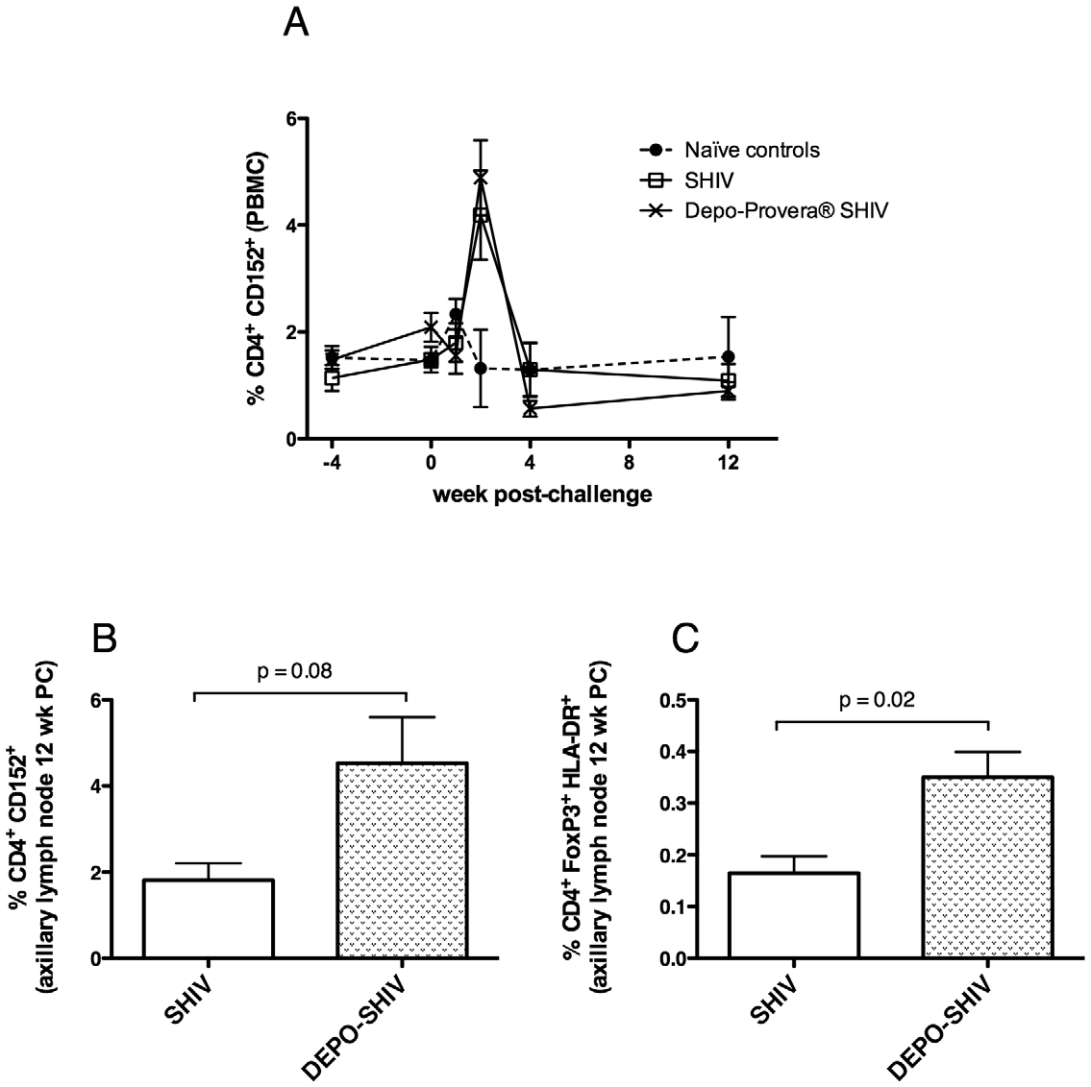
Frequencies of regulatory CD4^+^ T cells after progesterone treatment and IV SIVmac239 challenge. A) Percentages of CD4^+^ CD152^+^ T cells in PBMC. B) Percent of CD4^+^ FoxP3^+^ HLA-DR^+^ T cells after challenge in PBMC. C) Percentages of CD4^+^ CD152^+^ T cells in axillary LN of the vaccinated animals at 12 weeks post-challenge. D) Percentages of CD4^+^ FoxP3^+^ HLA-DR^+^ T cells in axillary LN of the vaccinated animals at 12 weeks post-challenge. (λ) Naïve control macaques (n = 7), (○) SHIV-immunized macaques (n = 5) and (5) Depo-Provera®-treated, SHIV-immunized macaques (n = 6).

CD4^+^ regulatory T cells were counted by staining for CD25, HLA-DR, CD152 and FoxP3. Both CD4^+^CD25^+^HLA-DR^+^ T cells and CD4^+^Foxp3^+^HLA-DR^+^ T cell frequencies were significantly higher on the day of challenge in the macaques treated with Depo-Provera® than in the untreated, SHIV-immunized macaques (*p = *0.047 and *p = *0.0018 respectively, T test, data not shown). Moreover, progesterone treatment increased the frequency of circulating CD4^+^CD152^+^ T cells between the day of treatment and the day of challenge (from 1.5% to 2.1%, *p = *0.08; Figure 6A). The frequency of CD4^+^CD152^+^ T cells was low and very stable after challenge in all groups, except for week 2 PC, when this subset increased in both SHIV-immunized groups (Figure 6A): however, this increase was significant only for the Depo-Provera®-treated group compared to the naïve controls (4.9% vs 2.3%, *p = *0.045). Additionally, in LNs of the progesterone-treated animals the frequency of CD4^+^CD152^+^ T cells tended to be higher than in the untreated, SHIV-immunized animals at 12 wk PC (4.53% vs. 1.81%; *p = *0.08; Figure 6B). There was also higher frequency of CD4^+^Foxp3^+^ HLA-DR^+^ T cells in the LNs of progesterone-treated animals compared to the untreated, SHIV-vaccinated animals (p = 0.02, Figure 6C).

### Depo-Provera® treatment reduced inflammatory responses after SIV challenge

**Figure 7:**
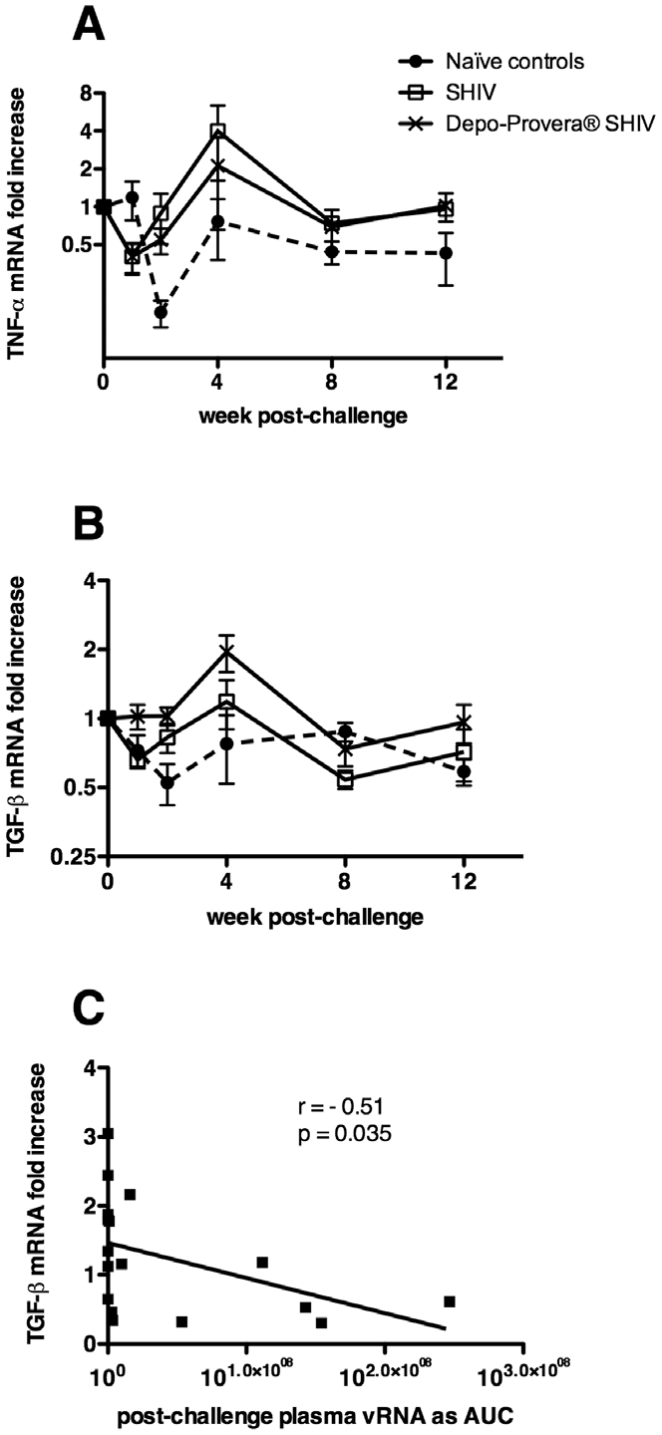
Cytokine mRNA levels after IV SIVmac239 challenge. Changes in gene expression are shown as the fold-change relative to the levels on the day of challenge for (A) TNF-α and (B) TGF-β. (C) Spearman rank correlation of TGF-β at 4 weeks PC, for all animals, with plasma viral RNA AUC between 1 and 20 weeks PC. (λ) Naïve control macaques (n = 7), (○) SHIV-immunized macaques (n = 5) and (5) Depo-Provera®-treated, SHIV-immunized macaques (n = 6).

After challenge, the Depo-SHIV and SHIV-immunized monkeys down-regulated TNF-α gene expression in PBMC compared to levels on the day of challenge. At week 1 PC, this decrease in TNF-α mRNA in both groups was highly significant (*p = *0.0005 and *p = *0.001 respectively, Figure 7A). Plasma TNF-α protein levels were low and variable in all of the monkeys before SIV challenge. However at 1 week PC, there were significantly lower plasma TNF-α levels in the progesterone-treated animals (2.1±0.6 pg/ml) compared to the untreated, SHIV group (5.1±1.2 pg/ml; *p = *0.05, T test).

In the Depo-Provera®-treated, SHIV group TGF-β gene expression was induced in PBMC (2-fold increase, *p = *0.035) 4 weeks after SIVmac239 challenge and then returned to basal levels rapidly (Figure 7B). TGF-β mRNA was also down regulated immediately after challenge, but only in the SHIV-immunized and the naïve control groups (*p = *0.0002 and *p = *0.03 respectively, Figure 7B). In fact, TGF-β mRNA levels were significantly higher in the PBMC of the progesterone-treated animals compared to the untreated, SHIV-immunized group at 1 week PC (*p = *0.046; Figure 7B), and compared to naïve controls at 2 and 4 week PC (*p = *0.008 and *p = *0.021; Figure 7B). Additionally, TGF-β mRNA levels at 4 weeks PC negatively correlated with plasma vRNA AUC levels from week 1 to 20 PC (*r* = −0.51, *P = *0.035, n = 17 x-y pairs, Figure 7C). These results suggest that Depo-Provera® treatment blunted the pro-inflammatory response to SIV challenge and enhanced T regulatory-mediated suppression of immune activation.

## Discussion

In the present study we found that Depo-Provera® did not blunt or abrogate control of viral replication after IV SIV challenge of SHIV-immunized female macaques. Thus, the abrogation of vaccine-induced protection by treatment with Depo-Provera® before vaginal SIV challenge is not due to systemic, immunologic effects of progestins. In addition, the possibly increased protection in progesterone-treated, immunized monkeys against IV SIV challenge is likely due to systemic effects of progestins on antiviral immunity. In fact SHIV immunized female macaques treated with Depo-Provera® developed a stronger and more polyfunctional CD8^+^ T cell response than untreated SHIV-immunized animals. Moreover, IL-2 secretion was much more abundant in SIV-specific CD8^+^ T cells from the Depo-Provera® SHIV-immunized group than in the SHIV-immunized untreated group. Thus, as previously suggested [24], the detection of circulating, SIV-specific polyfunctional CD8^+^ T cells secreting IL-2 may indicate an effective antiviral CD8^+^ T cell response. However, the route of virus infection may influence the effect of Depo-Provera® on antiviral CD8^+^ T cells responses. In contrast to the results reported here, we previously reported that, after vaginal SIV challenge, SHIV-immunized rhesus macaques that were treated with Depo-Provera® have weaker CD8^+^ T cell responses and less control of challenge virus replication than untreated, SHIV-immunized animals [4]. Further, other labs have also shown that Depo-Provera® supresses cellular immunity in monkeys and mice [13], [14].

Depo-Provera® treatment induced both transient T cell activation and an increase in T regulatory cell frequency, changes that may be critical for preventing aberrant immune activation after challenge [25]. The lack of disease progression in SIV-infected African green monkeys is associated with early expression of TGF-β [26] that presumably contributes to minimal immune activation and lack of disease progression in this natural host of SIV. Thus, a critical factor in the lack of disease progression in Depo-Provera® SHIV animals may be control of early T cell activation to avoid the aberrant immune activation that generally develops soon after SIV infection of macaques or HIV infection of humans [26], [27]. In contrast to the effect on female rhesus macaques, progesterone treatment decreased protection from IV challenge with SIVmac239 in SHIV-immunized, male rhesus macaques [21]. Although T cell activation was also enhanced in progesterone-treated males [21], the anti-inflammatory response that was found in females was not found in males. This gender-dependent difference in the effect of Depo-Provera® requires further study.

In summary, Depo-Provera® did not abrogate the control of viral replication but it may have improved viral control after IV challenge in SHIV-immunized female rhesus macaques. Depo-Provera® stimulated T cell activation and T regulatory responses before and just after SIV challenge. Further, Depo-Provera® enhanced TGF-**β** expression and decreased TNF-α secretion after challenge, establishing an anti-inflammatory environment in the treated animals. These changes correlated with enhanced control of challenge virus replication. Although it is possible that a direct effect of progesterone on viral replication at the cellular level contributed to the benefit observed, our findings clearly show that exogenous progestins do not have a detrimental impact on protection from IV challenge in female, SHIV-immunized macaques. Further, the decreased protection from vaginal SIV challenge in Depo-Provera® treated, SHIV-immunized animals must be due to local changes in the genital tract that enhance SIV transmission, such as thinning of the vaginal epithelium [9] or other immune effects limited to the female reproductive tract [10]. As hormonal contraceptive treatment is associated with disease progression in HIV-infected, untreated women [7], there is a clear and urgent need to understand how contraceptive compounds modulate the immunological response in the context of HIV infection and vaccination [8]. This study emphasizes the complex impact of sex steroids on antiviral immunity and vaccine-mediated protection from AIDS virus exposure.
